# Continuous Topical Oxygen Therapy Is Associated with Accelerated Nipple–Areolar Complex Necrosis Healing Following Nipple-Sparing Mastectomy: A Propensity-Matched Three-Way Comparison

**DOI:** 10.3390/cancers18121907

**Published:** 2026-06-11

**Authors:** Hyung-Suk Yi, Ho-Young Im, Jin-Hyung Park, Sung-Ui Jung, Jin-Hyuk Choi, Ku-Sang Kim, Yoon-Soo Kim

**Affiliations:** 1 Department of Plastic and Reconstructive Surgery, Kosin University Gospel Hospital, Kosin University College of Medicine, Busan 49267, Republic of Korea; sencha21@naver.com (H.-S.Y.); myhoyoung000@naver.com (H.-Y.I.); atreyue@naver.com (J.-H.P.); 2 Department of Surgery, Kosin University Gospel Hospital, Kosin University College of Medicine, Busan 49267, Republic of Korea; ist2000good@daum.net (S.-U.J.); drchoijinhyuk@gmail.com (J.-H.C.); ideakims@gmail.com (K.-S.K.)

**Keywords:** nipple-sparing mastectomy, nipple–areolar complex, topical oxygen therapy, hyperbaric oxygenation, breast reconstruction, propensity score matching, restricted mean survival time

## Abstract

Background/Objectives: Nipple–areolar complex (NAC) necrosis following nipple-sparing mastectomy (NSM) delays adjuvant therapy and compromises oncological outcomes. This retrospective propensity-matched cohort study compared continuous topical oxygen therapy (cTOT), hyperbaric oxygen therapy (HBOT), and standard care (SOC) in patients with NAC necrosis after NSM. Methods: Between January 2020 and August 2025, 213 patients with NAC necrosis after NSM at a single academic center met eligibility criteria (cTOT, *n* = 57; HBOT, *n* = 59; SOC, *n* = 97). Propensity score matching (1:1:1, 15 covariates, all standardized mean differences < 0.1) yielded 57 matched triplets (171 patients). The primary outcome was time to complete epithelialization, with restricted mean survival time (RMST) at 56 days designated as a co-primary metric. Results: cTOT was associated with the fastest healing (mean 30.5 ± 9.9 days vs. HBOT 36.4 ± 9.0, *p* = 0.007, vs. SOC 45.1 ± 10.6, *p* < 0.001). RMST analysis demonstrated a mean gain of 14.0 unhealed days (95% CI, 10.5–17.5) for cTOT versus SOC. Eight-week healing rates were 98.2% (cTOT), 96.5% (HBOT), and 87.7% (SOC). Multivariable Cox regression identified cTOT as an independent predictor of accelerated healing (HR 4.61; 95% CI, 2.99–7.11; *p* < 0.001), with results stable across incision-type and ptosis-grade sensitivity analyses. The treatment association was most pronounced in larger wounds (≥2.0 cm^2^; HR 6.85; *p* for interaction = 0.018). cTOT patients also reported significantly lower pain and higher satisfaction, with no device-related discontinuations (0/57). Conclusions: cTOT was associated with significantly accelerated NAC necrosis healing and, as a portable home-based therapy, may support oncological treatment timelines after NSM. The shorter time to adjuvant initiation observed for cTOT (continuous outcome) was not paralleled by a significant reduction in the >6-week delay endpoint, which requires confirmation in multicenter randomized trials.

## Simple Summary

Delayed healing of the nipple and surrounding pigmented skin after nipple-sparing mastectomy can postpone additional cancer treatment and increase physical and emotional stress for patients. This study compared three commonly used treatments for this complication: continuous topical oxygen therapy (cTOT), hyperbaric oxygen therapy, and standard wound care. In a propensity-matched analysis, cTOT was associated with the fastest healing, very high 8-week closure rates, lower pain, and greater patient satisfaction. The benefit was especially marked in larger wounds. Although the time to starting adjuvant therapy was shortened on average, the proportion of patients with a clinically defined delay (>6 weeks) was not significantly different and requires confirmation in adequately powered trials. Because cTOT is portable and can be used at home, it may help reduce disruption to postoperative cancer care. These findings support further multicenter randomized trials.

## 1. Introduction

Nipple-sparing mastectomy (NSM) has become the preferred approach for both therapeutic and risk-reducing mastectomy, with adoption rates reaching 20–40% at high-volume centers [1,2]. While NSM offers superior aesthetic and psychological outcomes [3], nipple–areolar complex (NAC) necrosis remains its most consequential complication, occurring in 5–30% of cases [4,5]. NAC necrosis is not merely a reconstructive complication—it delays adjuvant therapy. Wound healing delays postpone adjuvant chemotherapy beyond the recommended 6-week threshold [6]; delays exceeding 12 weeks are associated with diminished relapse-free survival (HR 1.6; 95% CI, 1.2–2.3) [7]. Meta-analytic data suggest that each 4-week delay is associated with a 6–15% increase in all-cause mortality [8]. When severe, NAC necrosis necessitates secondary surgery, further displacing the chemotherapy start date [9].

Current management options are suboptimal. Standard care—serial debridement with petroleum-based dressings—yields unpredictable healing, with variable outcomes and healing timelines that remain poorly defined [1,5]. Hyperbaric oxygen therapy (HBOT) stimulates neovascularization [10,11] but requires 2–4 hospital sessions, and its treatment burden can itself delay chemotherapy [12,13]. Continuous topical oxygen therapy (cTOT) is a portable, battery-powered system that delivers pure humidified oxygen 24/7 directly to the wound surface, leveraging oxygen’s established role as a cofactor for collagen synthesis, neovascularization, and antimicrobial activity [14,15,16]. A multicenter randomized controlled trial demonstrated its efficacy in diabetic foot ulcers (44.4% vs. 28.1% healing, *p* = 0.044) [17], yet no study has evaluated this modality for post-mastectomy NAC necrosis. Three treatment options are in clinical use for NAC necrosis, but no comparative effectiveness data exist to guide selection.

This study provides the first three-way comparison of cTOT, HBOT, and standard care for NAC necrosis following NSM. We hypothesized that continuous topical oxygen delivery would be associated with significantly accelerated healing compared with standard care (HR > 3.0) and demonstrate non-inferiority to HBOT. The primary objective was to compare time to complete epithelialization using propensity score matching (PSM) across 15 baseline covariates. Secondary objectives included evaluation of 8-week healing rates, adjuvant therapy delay, secondary surgery requirements, and identification of subgroups deriving the greatest benefit.

## 2. Materials and Methods

### 2.1. Study Design and Setting

This retrospective comparative cohort study was conducted at a single academic tertiary referral center in South Korea. All consecutive patients who developed NAC necrosis following NSM with immediate implant-based reconstruction between January 2020 and August 2025 were identified from a prospectively maintained database. Patients received cTOT, HBOT, or standard wound care (SOC) based on temporal availability, surgeon preference, and resource availability. The study was approved by the Institutional Review Board of Kosin University Gospel Hospital (Protocol Code 2025-09-022; date of approval: 15 October 2025), with a waiver of individual informed consent due to the retrospective design. Separate written informed consent was obtained for publication of clinical photographs. Reporting adhered to the STROBE guidelines [18].

### 2.2. Study Population

During the 6-year study period, 1043 patients at our institution underwent NSM with immediate implant-based reconstruction (the inclusion criterion frame for this study); of these, 318 developed clinically diagnosed NAC necrosis of any Dent grade, yielding an institutional NAC necrosis incidence of 30.5%.

**Figure 1:**
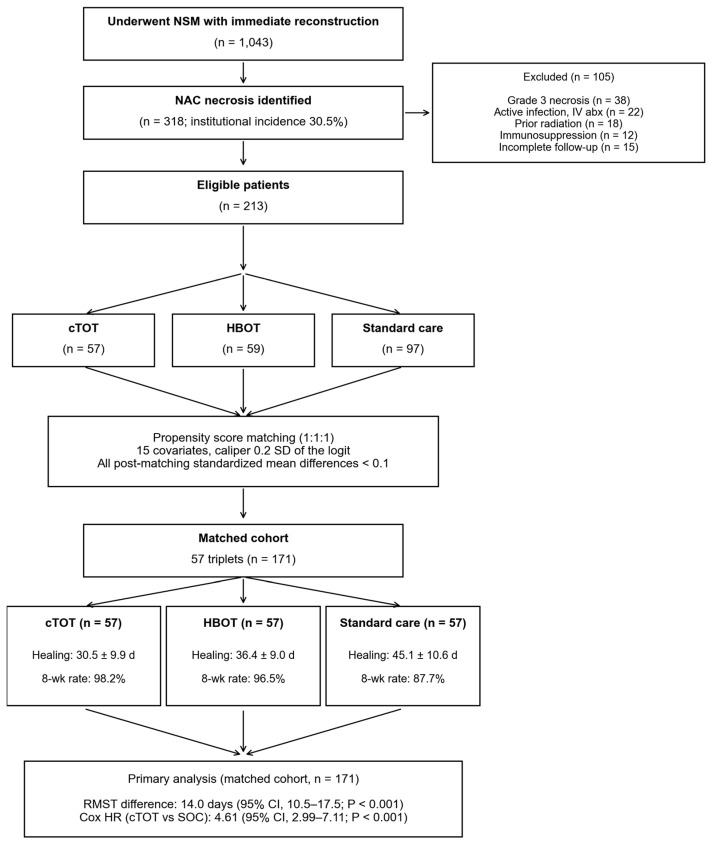
Patient flow diagram (STROBE). Of 318 patients screened, 105 were excluded. The final cohort comprised 213 patients (cTOT 57, HBOT 59, SOC 97). Propensity score matching (1:1:1) yielded 57 triplets (171 patients); all 15 covariates achieved SMD < 0.1.

All 318 NAC necrosis patients identified during the study period (Grade 1–3, including partial-thickness epidermolysis) were screened for the present analysis. Of these, 213 met eligibility criteria after exclusion of Grade 3 (full-thickness) necrosis (*n* = 38), active infection requiring intravenous antibiotics (*n* = 22), prior ipsilateral radiation (*n* = 18), immunosuppressive therapy (*n* = 12), and incomplete follow-up (*n* = 15) (Figure 1). Inclusion criteria were: female patients aged ≥ 18 years; NSM with immediate implant-based reconstruction using acellular dermal matrix (ADM); clinically diagnosed NAC necrosis of Grade 1 (partial-thickness, <50% surface involvement) or Grade 2 (≥50%) within 14 postoperative days (POD); and minimum 12-week follow-up. The final cohort comprised 57 cTOT, 59 HBOT, and 97 SOC patients.

### 2.3. Treatment Protocols

cTOT. The cTOT system used in this study was the NATROX^®^ Wound Oxygen System (Inotec AMD Ltd., Letchworth Garden City, UK)—a portable electrochemical oxygen generator (~100 g) producing pure humidified oxygen (>98%) at ~15 mL/hour, delivered via silicone tubing to an oxygen distribution system (ODS) placed directly over the wound bed (Supplementary Figure S1) [17,19]. The ODS was covered with a silicone foam dressing (Mepilex Border Flex, Mölnlycke Health Care, Gothenburg, Sweden), and the entire construct was sealed with a transparent film dressing (Tegaderm™, 3M, St. Paul, MN, USA) to maintain the oxygen-enriched microenvironment beneath the foam. Oxygen was delivered continuously (24 h/day, 7 days/week). Dressings were changed every 3–4 days at outpatient visits. Patients managed the device independently at home. Device compliance was assessed indirectly through outpatient visit attendance (mean attendance rate 96.3%) and visual inspection of dressing integrity and ODS positioning at each visit; objective device-usage logging was not available during the study period.

HBOT. Sessions were administered in a monoplace chamber at 2.4 atmospheres absolute (ATA) for 90 min once daily. Treatment courses ranged from 2 to 4 sessions based on clinical response. The 2–4 session course reflects our institutional protocol for acute post-mastectomy ischemic complications and is intentionally shorter than the 20–40 session courses standard in chronic-wound HBOT (e.g., diabetic foot ulcers, radiation-induced injury); the shorter course is bounded by the practical requirement to complete oxygen therapy without delaying the adjuvant chemotherapy window. Between sessions, wounds were dressed with petroleum-impregnated gauze (vaseline gauze) as the contact layer, covered by Mepilex Border Flex. Dressings were changed daily.

Standard care. Weekly sharp debridement was performed without adjunctive oxygen therapy. Wounds were dressed with petroleum-impregnated gauze (vaseline gauze) as the contact layer, covered by Mepilex Border Flex. Dressings were changed daily.

Wound dressing standardization. A critical design feature of this study was the use of the same secondary dressing—Mepilex Border Flex (silicone foam with adhesive border)—across all three treatment arms. This semi-occlusive foam dressing provides a standardized moist wound healing microenvironment, ensuring that between-group differences in healing cannot be attributed to the occlusive or moisture-retentive properties of the dressing. The groups differed only in the contact layer placed directly over the wound bed: the cTOT ODS (delivering continuous pure oxygen) versus petroleum-impregnated gauze (an inert, non-adherent barrier). The additional transparent film (Tegaderm) applied over the Mepilex Border Flex in the cTOT group served exclusively to seal the oxygen-enriched microenvironment and had no direct wound contact. Dressing change frequency differed (every 3–4 days for cTOT vs. daily for HBOT and SOC), a difference that, if anything, would favor the comparator arms through more frequent wound assessment and debridement opportunities.

All groups received identical perioperative care. All NSM procedures were performed by three dedicated breast surgeons using a standardized technique (inframammary fold, vertical robotic-assisted, or radial incision approach with subpectoral or prepectoral implant placement and ADM reinforcement). Intraoperative perfusion assessment by indocyanine green (ICG) angiography was not routinely available during the study period and therefore could not be incorporated into the propensity model. Details of the surgical technique are provided in Supplementary Methods S1. Treatment was initiated therapeutically upon clinical diagnosis of NAC necrosis (Dent grade ≥ 1)—never prophylactically—as soon as any necrotic change was identified, typically at postoperative day (POD) 2–5 with a comparable mean across the three groups (cTOT 5.5 ± 1.5; HBOT 5.2 ± 1.3; SOC 5.4 ± 1.6; *p* = 0.52). Once a patient was assigned to a treatment modality, that modality was continued until complete epithelialization, secondary surgery, or the 8-week study endpoint. Crossover or sequential combination of oxygen-based therapies was not permitted as a routine practice. During the study period, only two SOC patients (1.2% of the SOC arm) were escalated to HBOT after persistent stagnation beyond week 3; no other crossovers (SOC→cTOT, HBOT→cTOT, cTOT→HBOT, or sequential HBOT then cTOT) occurred. These two patients were analyzed by their original SOC assignment in the intention-to-treat analysis and excluded in a pre-specified per-protocol sensitivity analysis (Section 3.6). Intraoperative flap thickness was not routinely measured with calibrated calipers during the study period; however, post hoc surgeon assessment in cases that subsequently developed NAC ischemia suggested a flap thickness on the order of 5 mm or less, consistent with the institutional oncological priority of minimizing residual glandular tissue. This methodological limitation precludes formal quantitative correlation between flap thickness and NAC necrosis within the present dataset. Among NSM procedures performed at our institution during the study period, the majority were performed via a robotic-assisted (da Vinci) approach (302 of 428 procedures, 70.6%), with the remainder performed using a conventional open technique (126 of 428, 29.4%), based on an audit of the institutional operating-room scheduling database (bilateral cases counted as two procedure sides); the proportion of robotic NSM rose from 0% in 2020 to 82.1% in 2025, reflecting the institutional transition toward robotic NSM that began in 2022; the specific approach was selected based on tumor location, breast volume, surgeon preference, and patient consent. Tumescent infiltration of the subdermal plane with a dilute lidocaine–epinephrine solution (1:100,000 epinephrine) was performed prior to dissection in both approaches. Superficial subcutaneous dissection was performed predominantly by sharp scissor dissection, with technique variation across operators; deep-plane hemostasis and dissection used an ultrasonic-bipolar integrated energy device (Thunderbeat, Olympus, Tokyo, Japan), and monopolar coagulation was used only sparingly to minimize thermal injury to the dermal vasculature of the overlying skin and NAC.

### 2.4. Outcome Measures

The primary outcome was time to complete epithelialization, defined as the interval from treatment initiation to 100% re-epithelialization confirmed by clinical assessment and digital planimetry at two consecutive visits. To address potential non-proportionality of hazards, restricted mean survival time (RMST) analysis at the 56-day (8-week) restriction time was designated as the co-primary metric, providing a model-free estimate of absolute treatment benefit. Secondary outcomes included: 8-week complete healing rate; adjuvant chemotherapy delay (>6 weeks post-surgery; per NCCN guidelines [6], DCIS patients were retained in the matched cohort denominator but were not eligible for chemotherapy delay ascertainment and therefore could not contribute to the numerator); secondary surgery rate; implant loss; wound infection; patient-reported pain (VAS, 0–10) at Week 2; and patient satisfaction (numeric rating, 1–10). Secondary surgery was defined as any unplanned return to the operating room for wound-related complications, including debridement under general anesthesia, wound revision, implant exchange, or implant removal; the decision to proceed to surgery was made by the treating plastic surgeon based on clinical judgment (failure of conservative management at ≥4 weeks, exposed implant/ADM, or progressive necrosis). An alternative composite adjuvant delay definition (healing time > 40 days and Week 4 wound area > 1.0 cm^2^) was evaluated as a pre-specified sensitivity analysis.

### 2.5. Wound Assessment

NAC necrosis was graded per Dent et al. [1]: Grade 1 (<50% NAC surface), Grade 2 (≥50%), and Grade 3 (full-thickness loss). Wound area was measured at baseline, Weeks 1, 2, and 4 using standardized digital planimetry (ImageJ software, two-point calibration) on photographs obtained with a Canon EOS 5D Mark IV (100 mm macro lens, ring flash) [20]. Measurements were performed by two blinded observers (inter-rater ICC = 0.94, 95% CI: 0.91–0.96); observer blinding applied to wound-area planimetry at the time of each measurement (observers were not informed of prior wound-area values), while treatment-arm masking was not feasible given the visible device differences across the cTOT, HBOT, and SOC arms. Percentage area reduction (PAR) was calculated at Weeks 2 and 4 as a surrogate marker for healing prognosis. The clinical photography and de-identification protocol is detailed in Supplementary Methods S2.

### 2.6. Propensity Score Matching

Generalized propensity scores were estimated using multinomial logistic regression incorporating 15 baseline covariates: age, BMI, smoking status, diabetes mellitus, hypertension, prior radiation, neoadjuvant chemotherapy, tumor location, tumor–nipple distance, cancer stage, mastectomy specimen weight, implant size, ADM type, necrosis grade, and initial necrosis area [21,22]. Matching was performed using a greedy nearest-neighbor algorithm (1:1:1, caliper 0.2 SD of the logit), yielding 57 matched triplets (171 patients). All covariates achieved SMD < 0.1 in the matched cohort (Table 1) [23]. We selected 1:1:1 matching over alternative approaches (inverse probability of treatment weighting [IPTW], overlap weighting) because: (i) it provides a transparent matched cohort amenable to direct clinical interpretation; (ii) the relatively similar group sizes and adequate propensity score overlap supported feasible matching; and (iii) post-match covariate balance was excellent. As a supplementary analysis, IPTW-weighted Cox regression yielded consistent estimates (Supplementary Tables S3 and S5).

### 2.7. Statistical Analysis

**Figure 2:**
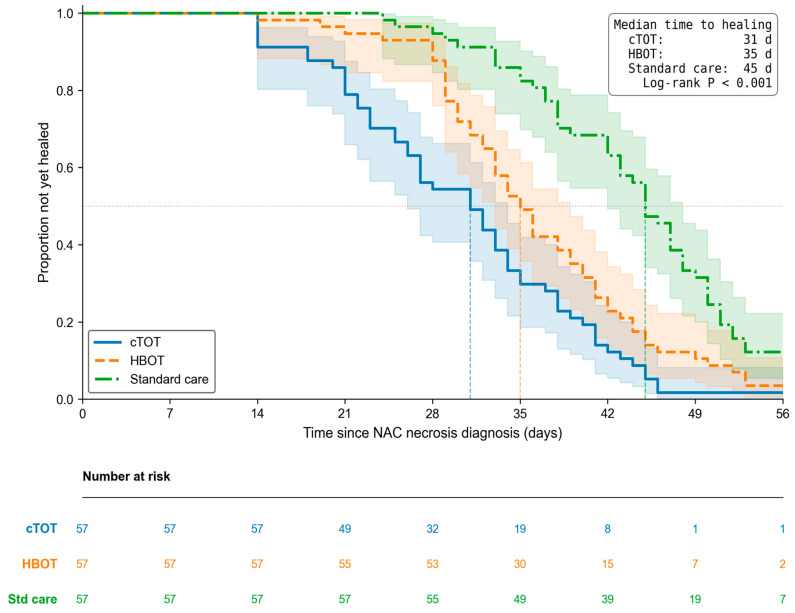
Kaplan–Meier curves for time to complete epithelialization (matched cohort, *n* = 171). cTOT (blue, solid), HBOT (orange, dashed), and SOC (green, dash-dot). Log-rank χ^2^ = 80.9, *p* < 0.001.

Time to complete epithelialization was analyzed using Cox proportional hazards regression. Patients were right-censored at the 8-week study endpoint or at the time of secondary surgery, whichever occurred first. Secondary surgery was modeled as a competing event in separate Fine–Gray subdistribution hazard and cause-specific hazard analyses (Supplementary Table S2, Supplementary Figure S2). Multivariable modeling used backward elimination (*p* < 0.10), robust (sandwich) variance estimators for matched clusters, and per-patient mean inter-visit interval (days) as a continuous covariate to address surveillance bias [22]. Unadjusted between-arm comparisons in the matched cohort are presented as group means with standard deviations (Table 2), Kaplan–Meier survival curves with log-rank pairwise tests (Figure 2), and 8-week event rates with χ^2^/Fisher exact tests, while the multivariable Cox model (Table 3) provides the confounder-adjusted hazard ratios. The proportional hazards assumption was verified using Schoenfeld residuals; model discrimination was assessed by the Harrell C-statistic. Unless otherwise stated, all primary and secondary outcome analyses were conducted in the matched cohort (*n* = 171); full-cohort results (*n* = 213) are presented in the Supplementary Materials for transparency.

Kaplan–Meier curves were compared by log-rank test with Bonferroni-adjusted pairwise comparisons (α = 0.017). Binary secondary endpoints were compared using chi-square or Fisher exact tests. Pre-specified subgroup analyses tested for effect modification by necrosis grade, wound area, BMI, smoking, diabetes, and cancer stage using interaction terms (*p* < 0.10 threshold).

Seven sensitivity analyses assessed robustness: (1) Rosenbaum bounds for unmeasured confounding; (2) E-values; (3) Fine–Gray competing risks analysis (secondary surgery as competing event); (4) interrupted time series analysis adjusting for era effects; (5) complete case analysis; (6) per-protocol analysis excluding treatment crossovers [24,25,26,27]; and (7) dressing change frequency sensitivity analysis, in which a binary covariate (every 3–4 days vs. daily dressing changes) was added to the multivariable Cox model to assess whether the reduced dressing change frequency in the cTOT group influenced the primary outcome estimate. The interrupted time series analysis incorporated enrollment order as a continuous covariate and a segmented regression structure, with the segmentation point at patient enrollment #155 (corresponding to cTOT introduction in September 2024). Model diagnostics included Durbin–Watson testing for autocorrelation and alternative specifications using calendar year and spline terms (Supplementary Methods S3). IPTW-weighted Cox regression was also performed to confirm that results were not sensitive to the choice of propensity score method (Supplementary Table S5). RMST analysis was performed at the 56-day (8-week) restriction time as a co-primary analysis to provide a model-free estimate of treatment benefit that does not depend on the proportional hazards assumption. All analyses were performed in R 4.3.0 (survival, MatchIt, cmprsk, segmented). A two-tailed *p* < 0.05 was considered significant.

Sample size. An a priori power analysis required 180 patients (60/group) for 80% power to detect HR 2.5 at α = 0.017 (Bonferroni-adjusted). The final matched sample of 171 (57/group) fell 9 patients short of this target; however, the observed treatment effect (HR 4.61) was more than twice the magnitude assumed at study design. The observed RMST 95% confidence interval (10.5–17.5 days) excluded the null; precision for the primary endpoint was therefore adequate despite the modest sample-size shortfall. Secondary binary endpoints (adjuvant delay > 6 weeks; secondary surgery) remained underpowered (post hoc power 27% and 41%, respectively) and are explicitly flagged as exploratory.

Missing data. The primary outcome (time to complete epithelialization) and all 15 propensity-matching covariates were available for 100% of the analytical cohort, as completeness of these variables was a pre-specified inclusion criterion. All reported secondary outcomes (8-week complete healing, adjuvant therapy delay, secondary surgery, implant loss, wound infection, Week-2 pain VAS, patient satisfaction, and serial wound-area measurements at Weeks 1, 2, and 4) were complete for every analyzed patient (0/171 missing in the matched cohort). Variables that were missing by design—ICG perfusion score (not routinely available), crossover documentation (only two SOC→HBOT crossovers), and the cTOT-specific burden questionnaire (administered only to the cTOT arm)—were not included in the primary analytical framework. All primary analyses were performed on complete cases without imputation, and the complete-case sensitivity analysis (Section 3.6) confirmed the robustness of findings.

## 3. Results

### 3.1. Patient Selection and Baseline Characteristics

Of 318 patients screened, 213 met eligibility criteria: 57 cTOT, 59 HBOT, and 97 SOC (Figure 1). Baseline characteristics of the full cohort (*n* = 213) were comparable across groups (Table 1). Tumor–nipple distance was the only variable reaching pre-matching significance (*p* = 0.023), and several standardized mean differences exceeded 0.1 (hypertension, SMD 0.478; tumor–nipple distance, 0.404; diabetes, 0.328), confirming the necessity of propensity score adjustment. Following 1:1:1 matching (57 triplets, 171 patients), all 15 covariates achieved SMD < 0.1. No treatment crossovers occurred between the cTOT and SOC groups; two SOC patients were escalated to HBOT during the study period and were analyzed per their original assignment in the intention-to-treat analysis, with a per-protocol sensitivity analysis excluding these patients (see Section 3.6). Median follow-up to the primary outcome event or 8-week censoring in the matched cohort was 37 days (interquartile range 30–45 days; range 14–56 days), corresponding to a total of 6330 person-days of observation (cTOT 1741; HBOT 2077; SOC 2512). All matched patients met the pre-specified 12-week minimum clinical follow-up requirement.

### 3.2. Primary Outcome (Matched Cohort, n = 171)

cTOT was associated with significantly faster healing than both comparators (Table 2). In the matched cohort, mean time to complete epithelialization was 30.5 ± 9.9 days for cTOT, 36.4 ± 9.0 for HBOT, and 45.1 ± 10.6 for SOC (*p* < 0.001), representing a 32.4% reduction relative to SOC. All pairwise comparisons were significant (cTOT vs. SOC, *p* < 0.001; cTOT vs. HBOT, *p* = 0.007; HBOT vs. SOC, *p* < 0.001). Kaplan–Meier curves (Figure 2) were separated by Week 2 (log-rank χ^2^ = 80.9, *p* < 0.001). Eight-week healing rates were 98.2% (cTOT), 96.5% (HBOT), and 87.7% (SOC). RMST analysis—designated as the co-primary metric—demonstrated 14.0 fewer unhealed days for cTOT compared with SOC (95% CI, 10.5–17.5; *p* < 0.001; Supplementary Figure S3), providing a clinically tangible, model-free estimate of absolute treatment benefit. Full-cohort results were consistent (Supplementary Table S6).

### 3.3. Wound Healing Trajectory (Matched Cohort)

**Figure 3:**
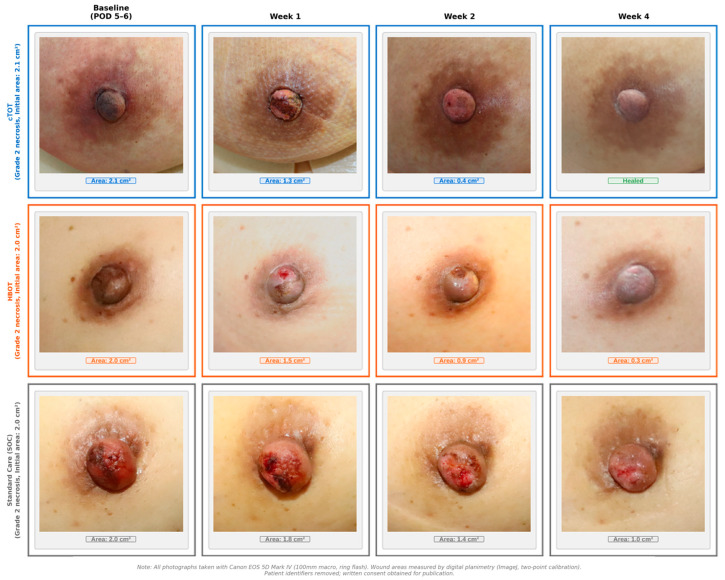
Representative clinical photographs of healing trajectories. The three patients displayed were selected from the matched cohort to illustrate typical healing-time progression in each treatment arm; the photographs are representative cases, not a complete sampling of cohort topography. All three displayed cases happen to show nipple-centered partial-thickness necrosis (NAC area involvement 8–17% of total areolar surface area), which corresponds to the predominant lesion pattern in this cohort (144/213 patients, 67.6%), but the broader cohort includes a substantial proportion of patients with extension into partial-areola (*n* = 59, 27.7%) and ≥50% NAC-extensive involvement (*n* = 10, 4.7%). Written informed consent was obtained for publication of clinical photographs; identifying features have been removed.

PAR at Week 2 was 45.4 ± 7.5% for cTOT, 41.0 ± 8.9% for HBOT, and 34.7 ± 8.2% for SOC (*p* < 0.001; Table 2). By Week 4, the majority of cTOT wounds had closed completely (median PAR 100%, residual area 0.00 cm^2^), whereas HBOT and SOC showed median residual areas of 0.32 and 1.14 cm^2^, respectively (Figure 3).

### 3.4. Secondary Outcomes (Matched Cohort)

Adjuvant therapy delay (>6 weeks) occurred in 5.3% of cTOT, 5.3% of HBOT, and 14.0% of SOC patients (*p* = 0.262; Table 2). When analyzed as a continuous outcome, median time from surgery to adjuvant therapy initiation was 35 days (IQR 28–42) for cTOT, 38 days (IQR 30–46) for HBOT, and 44 days (IQR 33–56) for SOC (*p* = 0.008). After adjustment for matched covariates, cTOT was associated with a mean reduction of 8.2 days (95% CI, 2.7–13.7; *p* = 0.004) in time to adjuvant therapy initiation compared with SOC. Reasons for delay were not systematically adjudicated; delays may have resulted from wound-related, systemic, or logistic factors. The binary endpoint did not reach statistical significance, and the study was not powered for this outcome (post hoc estimated power 27%; approximately 230 patients per group would be required for 80% power). The absolute risk reduction between cTOT and SOC was 8.7 percentage points (NNT = 12 to prevent one > 6-week adjuvant delay). The mechanistic interpretation of how accelerated wound healing relates to earlier adjuvant initiation is reserved for the Discussion.

Secondary surgery was required in 5.3%, 12.3%, and 19.3%, respectively (*p* = 0.132; NNT = 7 for cTOT vs. SOC). No cTOT patients experienced implant loss (vs. 1.8% HBOT, 1.8% SOC). HBOT-related adverse events included otic barotrauma (*n* = 4, 7.0%), claustrophobia requiring session modification (*n* = 3, 5.3%), and transient myopia (*n* = 2, 3.5%); two patients discontinued HBOT prematurely (both retained in the intention-to-treat primary analysis per assigned group). cTOT device-related events included mild periwound maceration (*n* = 3, 5.3%) and contact dermatitis at the film adhesive site (*n* = 2, 3.5%); none required treatment discontinuation (Supplementary Table S4). cTOT patients reported significantly lower pain (VAS 2.8 vs. 3.8 vs. 4.7; *p* < 0.001) and higher satisfaction (8.0 vs. 7.2 vs. 5.7; *p* < 0.001). Device wearability was qualitatively assessed during routine clinical follow-up. Across the 57-patient cTOT arm, no patient (0/57) discontinued therapy because of device-related burden, application difficulty, or portability inconvenience, and no formal wearability complaints were registered to clinical staff. Outpatient follow-up attendance was 96.3%. A structured wearability questionnaire was not administered prospectively during this retrospective period; one is planned for our prospective follow-up study.

### 3.5. Cox Regression and Subgroup Analysis (Matched Cohort)

Multivariable Cox regression identified a significant independent treatment association (Table 3). cTOT demonstrated an HR of 4.61 (95% CI, 2.99–7.11; *p* < 0.001) and HBOT an HR of 2.26 (95% CI, 1.51–3.38; *p* < 0.001), both relative to SOC. The cTOT-to-HBOT HR ratio was 2.04, corresponding to approximately twice the instantaneous healing probability. Necrosis grade (Grade 2: HR 2.08; *p* = 0.001) and initial wound area (HR 0.76 per cm^2^; *p* = 0.005) were significant independent predictors. The model demonstrated good discrimination (C-statistic = 0.72) with no global proportional hazards violation, although the cTOT variable showed a marginally significant time-dependent effect (Schoenfeld P = 0.022), consistent with a front-loaded benefit that attenuates as wounds approach complete healing. The co-primary RMST analysis, which does not depend on the proportional hazards assumption, confirmed the finding: the mean healing time gained with cTOT versus SOC was 14.0 days (95% CI, 10.5–17.5; *p* < 0.001).

**Figure 4:**
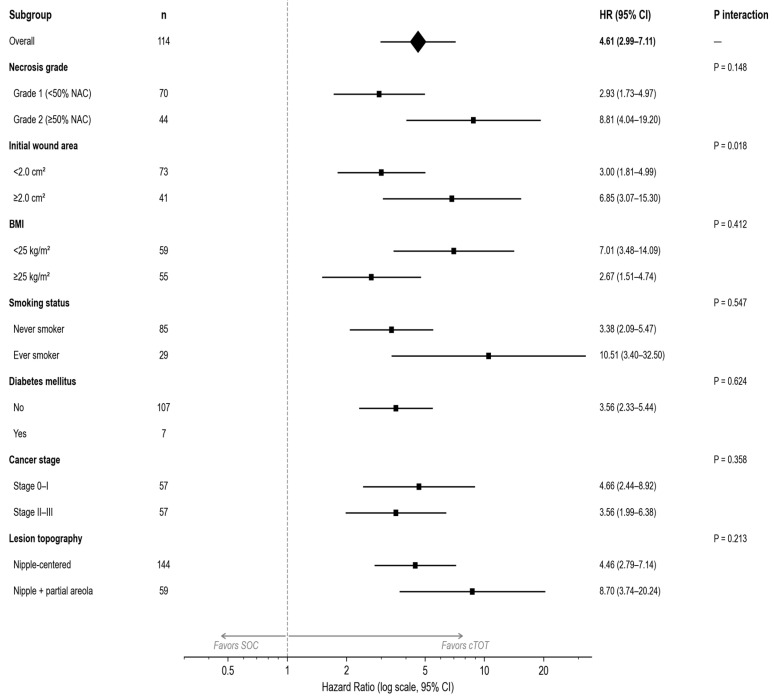
Forest plot of cTOT treatment association across subgroups. HR > 1.0 indicates faster healing. Most pronounced effect modification for wound area (*p* = 0.018; exploratory under Bonferroni).

Subgroup analyses (Table 4; Figure 4) demonstrated a consistent cTOT benefit across strata. The most pronounced treatment association was observed in larger wounds (≥2.0 cm^2^; HR 6.85 vs. 3.00 for smaller wounds; P for interaction = 0.018). Grade 2 necrosis patients showed a numerically greater benefit (HR 8.81 vs. 2.93 for Grade 1; P for interaction = 0.148). Given six subgroup comparisons, the wound–area interaction (*p* = 0.018) was the most pronounced but does not formally survive strict Bonferroni correction (α = 0.008); all subgroup findings should be considered exploratory. Small numbers of current smokers (*n* = 11) and diabetic patients (*n* = 14) precluded reliable subgroup estimation. Lesion topography subgrouping (Figure 4) likewise yielded cTOT hazard ratios of 4.46 (95% CI 2.79–7.14) for nipple-centered lesions and 8.70 (95% CI 3.74–20.24) for nipple plus partial-areola lesions, consistent with the wound–area interaction; the NAC-extensive subgroup was too small for reliable estimation. The cTOT applicator (NATROX^®^ ODS) has an active surface diameter of approximately 4.5 cm (~16 cm^2^ active surface area), providing single-placement coverage across the full range of initial wound sizes observed in this cohort.

### 3.6. Sensitivity Analyses

All seven pre-specified sensitivity analyses were consistent with the primary findings and supported the primary findings (Supplementary Table S1). E-values were 9.70 (point estimate) and 6.22 (lower CI). In practical terms, to fully explain the observed cTOT–healing association through unmeasured confounding alone, a hypothetical confounder would need to be associated with both the probability of receiving cTOT and the probability of healing by at least 6.2-fold each—a magnitude exceeding established wound healing confounders such as smoking (RR~2.0) or diabetes (RR~1.5). While E-values quantify the plausibility rather than the probability of confounding, they indicate that the observed association is unlikely to be entirely attributable to unmeasured bias. Rosenbaum bounds demonstrated significance up to Γ = 24, meaning that an unmeasured confounder would need to increase the odds of treatment assignment by 24-fold to overturn the result—a threshold exceeding all known clinical confounders in breast reconstruction. Cause-specific (HR 4.35), per-protocol (HR 4.64), and era-adjusted interrupted time series (HR 5.02; enrollment order *p* = 0.752) analyses all yielded consistent estimates (Supplementary Figure S4). The dressing change frequency sensitivity analysis, adjusting for the difference in dressing change intervals (every 3–4 days for cTOT vs. daily for comparators), yielded a virtually unchanged cTOT HR of 5.03 (95% CI, 3.31–7.64; *p* < 0.001), confirming that the treatment association was not confounded by dressing change frequency. Additional multivariable Cox analyses incorporating incision type, ptosis grade, mastectomy flap thickness, and NAC involvement percentage as covariates produced no statistically significant change in the cTOT treatment association; the result was robust to these surgical–anatomic confounders.

## 4. Discussion

This propensity-matched cohort study of 213 patients provides the first three-way comparison of cTOT, HBOT, and standard care for NAC necrosis following NSM. cTOT was associated with significantly accelerated wound healing, with a mean time to complete epithelialization of 30.5 days versus 36.4 for HBOT and 45.1 for SOC, an independent association in multivariable Cox regression (HR 4.61; 95% CI, 2.99–7.11; *p* < 0.001), and a co-primary RMST gain of 14.0 days (95% CI, 10.5–17.5). Subgroup analysis revealed the most pronounced effect modification with initial wound area (*p* = 0.018; HR 6.85 for lesions ≥ 2.0 cm^2^). Secondary outcomes—including the binary adjuvant chemotherapy delay endpoint (5.3% vs. 14.0%; *p* = 0.262) and secondary surgery rate (5.3% vs. 19.3%; *p* = 0.132)—did not reach statistical significance for the binary comparisons, but the continuous outcome showed an 8.2-day reduction in time to adjuvant initiation (*p* = 0.004), and the consistent direction of effect across endpoints suggests that accelerated healing may translate into clinically meaningful benefits requiring confirmation in adequately powered trials.

Continuous versus intermittent oxygenation. The cTOT advantage over HBOT (HR ratio 2.04) may reflect the pharmacokinetics of wound oxygenation. cTOT delivers pure humidified oxygen continuously to the wound surface, whereas HBOT provides supraphysiological oxygen for 90–120 min per session followed by ~22 h of relative hypoxia [15]. Sustained oxygen exposure supports continuous angiogenesis, collagen crosslinking, and antimicrobial activity, processes that may be interrupted between HBOT sessions [15,16]. This hypothesis is consistent with our wound trajectory data: Kaplan–Meier curves diverged by week 2, and cTOT achieved a median PAR of 100% by week 4 versus 80.2% for HBOT. The marginal Schoenfeld P (0.022) for cTOT further suggests a front-loaded benefit consistent with accelerated early-phase healing. Mechanistic confirmation will require prospective transcutaneous oxygen monitoring. We further note that the observed cTOT-to-HBOT HR ratio of 2.04 should not be interpreted as a pure ‘continuous vs. intermittent’ biological comparison; it also reflects a substantial cumulative oxygen-dose differential—cTOT delivers 24 h continuous oxygen throughout the entire treatment period, whereas HBOT in our institutional protocol provides 2–4 brief sessions totaling approximately 3–6 h of supraphysiological exposure. The cTOT-vs.-HBOT comparison therefore conflates three distinct axes—local vs. systemic delivery, continuous vs. intermittent exposure, and cumulative oxygen dose—that future mechanistic studies should disentangle through randomized factorial designs with equivalent cumulative-dose arms.

Magnitude of treatment effect and the role of RMST. The observed HR of 4.61 exceeds typical wound-management effect sizes and warrants cautious interpretation. We recommend the RMST difference of 14.0 days (95% CI, 10.5–17.5) as the primary clinical metric: it is model-free, robust to the marginal non-proportionality observed for cTOT (Schoenfeld *p* = 0.022), and provides an absolute, clinically tangible estimate. The HR magnitude is biologically plausible when the comparison is considered: cTOT delivers continuous pure oxygen directly to ischemic tissue 24 h/day, whereas SOC provides no targeted oxygen supplementation—a mechanistic on/off comparison rather than a dose–response between two active therapies. Standardized Mepilex Border Flex dressing across all three arms excludes dressing-related confounding as an alternative explanation. The magnitude is also consistent with the Serena randomized trial, which showed a 58% relative healing improvement for cTOT in diabetic foot ulcers [17]—a more challenging wound population than post-surgical NAC necrosis. Bounding the effect explicitly against the chronic-wound cTOT literature is informative: the Serena RCT corresponds to an odds ratio of approximately 2.0 for healing at 12 weeks [17]. Our observed Cox HR of 4.61 in acute post-NSM ischemic necrosis is therefore approximately twice the magnitude seen in chronic-wound trials. We attribute this gap to biological differences—acute ischemic tissue retains angiogenic potential and responds rapidly to restored oxygenation, whereas chronic-wound tissue has a senescent cellular environment that requires prolonged restoration. We acknowledge, however, that if residual era or selection confounding partially inflates the observed effect, the true cTOT effect may be modestly smaller than the observed HR 4.61 yet is unlikely to fall below a clinically meaningful magnitude—which would still justify, rather than diminish, the proposed multicenter randomized trial.

Oncological context. The timing of adjuvant chemotherapy is a well-established prognostic determinant: delays exceeding 12 weeks are associated with diminished relapse-free survival (HR 1.6; 95% CI, 1.2–2.3) [7], and each additional 4-week delay confers a 6–15% increase in all-cause mortality [8]. NAC necrosis prolongs healing and, when severe, mandates secondary surgery that further displaces chemotherapy initiation. In our cohort, adjuvant delay > 6 weeks occurred in 5.3% of cTOT, 5.3% of HBOT, and 14.0% of SOC patients (*p* = 0.262). The non-significant binary result reflects low absolute event rates, inadequate statistical power for this secondary endpoint, and absence of cause-specific delay adjudication. We emphasize that the pre-specified binary endpoint (>6-week delay per NCCN guidelines [6]) represents the clinically meaningful threshold and was negative for between-group comparison. The continuous time-to-adjuvant outcome—a pre-specified secondary analysis, not a replacement primary—yielded a significant 8.2-day reduction (95% CI, 2.7–13.7; *p* = 0.004) for cTOT vs. SOC after covariate adjustment, providing supportive and hypothesis-generating evidence that accelerated wound healing may translate into earlier adjuvant access; this finding requires confirmation as a primary endpoint in an adequately powered prospective trial.

Secondary surgery data support a downstream benefit of accelerated healing. cTOT patients required reoperation in 5.3% of cases compared with 12.3% for HBOT and 19.3% for SOC (absolute risk difference 14.0 pp; NNT = 7), though *p* = 0.132. Implant loss occurred in 0% (cTOT) vs. 1.8% (HBOT) vs. 1.8% (SOC). Faster wound closure may reduce vulnerability to infection, implant exposure, and the delays that secondary procedures impose. The absolute SOC reoperation rate (19.3%) appears higher than commonly reported NSM series and likely reflects the same institutional thin-flap policy that underlies our 30.5% NAC necrosis incidence—a deliberate oncological trade-off that maximizes residual-tissue clearance at the cost of an ischemic-complication burden that, in the absence of effective adjunctive oxygen therapy, more frequently progresses to surgical revision. Regarding the role of mesh: all reconstructions in this cohort used acellular dermal matrix (ADM) without synthetic mesh, and ADM type was included as a propensity-matching covariate; we therefore cannot directly compare ADM-only versus synthetic-mesh or hybrid ADM-plus-synthetic constructs, which have been reported to differ in seroma, capsular contracture, and infection profiles. Comparative evaluation of mesh material in the setting of NAC ischemia represents an important direction for future investigation. The cTOT-associated 14 percentage-point absolute reduction in reoperation (5.3% vs. 19.3%; NNT = 7) did not reach statistical significance for the binary endpoint (*p* = 0.132; post hoc power 41%), but the direction and magnitude are clinically meaningful and converge with the primary healing-time finding.

Effect modification and clinical targeting. Initial wound area was the most pronounced effect modifier (P for interaction = 0.018): wounds ≥ 2.0 cm^2^ showed HR 6.85 (95% CI 3.07–15.30) versus 3.00 for smaller wounds. The wide CI for the larger-wound subgroup reflects limited sample size and indicates a qualitatively greater benefit rather than a precise point estimate. Of six pre-specified subgroup comparisons, the wound–area interaction (*p* = 0.018) was the most pronounced but does not formally survive strict Bonferroni correction (α = 0.008); all subgroup findings are exploratory. Grade 2 necrosis showed a numerically greater effect (HR 8.81 vs. 2.93 for Grade 1; *p* interaction = 0.148). These findings support a wound-size-stratified algorithm: cTOT is the preferred therapy for lesions ≥ 2.0 cm^2^, where the HR difference vs. HBOT widens most clearly. The cTOT applicator diameter (~4.5 cm) further supports its use in this subgroup by enabling adequate coverage of larger NAC lesions with a single placement, without requiring repositioning or multiple applicators.

Comparison with existing literature. Evidence for oxygen-based therapies in NAC necrosis is limited. Shuck et al. found no significant difference between HBOT and SOC in 13 patients (*p* = 0.15) [12], and Idris et al.’s systematic review identified only seven studies (63 patients, no RCTs) [13]. More recently, Shen et al. reported 100% wound closure in 20 patients treated with HBOT for complex post-mastectomy wounds (median 30.5 sessions, 65% with prior radiation), with earlier HBOT initiation associated with faster healing [28]. With 213 patients—exceeding the combined sample of all prior HBOT studies for this indication—this cohort provides the largest controlled evidence base to date for the management of NAC necrosis after NSM [13]. The HBOT HR of 2.26 (95% CI 1.51–3.38) validates HBOT’s benefit over SOC. We acknowledge HBOT’s mechanistic advantages not shared by topical oxygen (systemic dissolved plasma oxygen, bactericidal supraphysiological pressures, established evidence for compromised flaps and radiation injury [10,11]). These findings do not diminish HBOT’s role where cTOT is unavailable but introduce an alternative with complementary practical advantages.

In chronic wounds, Serena et al. demonstrated 44.4% healing at 12 weeks for cTOT vs. 28.1% for SOC in diabetic foot ulcers (*p* = 0.044) [17], and Molina et al. reported 64% healing in hard-to-heal wounds [29]. The substantially larger effect size in our study (HR 4.61 vs. ~HR 2.0–2.5 in chronic wound trials) likely reflects different pathophysiology: acute post-surgical ischemic necrosis involves tissue with preserved angiogenic potential that responds rapidly to restored oxygenation, unlike the senescent cellular environment of chronic wounds.

Practical advantages. As a portable, battery-powered device (~100 g) carried in a belt pouch, cTOT enables home-based therapy with outpatient follow-up only, in contrast to HBOT, which requires 2–4 visits to a specialized hyperbaric chamber per treatment course—particularly burdensome for patients distant from HBOT-equipped centers. This access difference is reflected in lower pain scores (VAS 2.8 vs. 3.8 vs. 4.7; *p* < 0.001) and higher satisfaction (8.0 vs. 7.2 vs. 5.7; *p* < 0.001) for cTOT, and in the absence of any treatment discontinuation in our 57-patient cTOT cohort. The satisfaction differential likely captures access convenience and treatment-related burden—cTOT is administered at home whereas HBOT requires hospital chamber visits and SOC requires weekly outpatient debridement—rather than satisfaction with healing outcome per se; a treatment-blinded wound-specific patient-reported outcome (e.g., BREAST-Q wound domain) would be required for direct outcome-quality comparison. cTOT also avoids the absolute and relative contraindications associated with hyperbaric exposure—uncontrolled bullous pulmonary disease, certain chemotherapeutic agents (e.g., doxorubicin, bleomycin), claustrophobia, untreated otologic disease, and pneumothorax—expanding the patient population eligible for oxygen-based therapy.

Robustness of findings. Several design features support internal validity: propensity score matching on 15 covariates (all SMD < 0.1), standardized Mepilex Border Flex dressing across all arms (isolating oxygen delivery as the principal between-group variable), and seven pre-specified sensitivity analyses—including E-value (9.70; lower CI 6.22), Rosenbaum Γ = 24, interrupted time series (era-adjusted HR 5.02), per-protocol (HR 4.64), Fine–Gray competing risks (HR 4.35), IPTW-weighted (HR 4.88), and dressing-frequency-adjusted (HR 5.03) models (Section 3.6; Supplementary Table S1). The convergence of these independent approaches—each addressing a distinct bias source—supports the inference that residual confounding is an unlikely sole explanation for the observed association, though definitive causal attribution requires randomized evidence.

Limitations. Although the matched cohort (*n* = 171) was nine patients short of the a priori target (*n* = 180), the observed RMST 95% confidence interval (10.5–17.5 days) and Cox HR 95% confidence interval (2.99–7.11) both excluded the null with wide margins; precision for the primary endpoint was therefore adequate despite the modest sample-size shortfall. Secondary binary endpoints—adjuvant delay > 6 weeks (post hoc power 27%) and secondary surgery (41%)—remained underpowered. The retrospective single-center design is the principal limitation: despite 15-covariate matching, residual confounding by surgeon preference, evolving wound-care protocols, and patient self-selection cannot be excluded. We further note that PSM and the E-value/Rosenbaum analyses quantify residual confounding by measured and unmeasured baseline covariates; selection bias arising from the surgeon-preference and resource-availability components of treatment assignment is a structurally distinct residual concern that is not directly bounded by these tools and that only a randomized trial can fully resolve. The chronological adoption of cTOT introduces potential era effects, although the interrupted time series analysis (enrollment order *p* = 0.752), Rosenbaum bounds (Γ = 24), and E-value (lower CI 6.22) provide reassurance. Two SOC patients escalated to HBOT during the study; both were analyzed by their original assignment, with per-protocol sensitivity confirming results (HR 4.64). The standardized Mepilex Border Flex dressing across all arms mitigates dressing-related confounding; the only residual differences—the cTOT ODS contact layer (which directly reflects the intervention under study) and dressing change frequency (every 3–4 days for cTOT vs. daily for comparators, with adjustment yielding HR 5.03)—do not provide an alternative explanation for the treatment effect.

Other limitations. Objective oxygenation biomarkers (TcPO_2_, ICG angiography) were not available and preclude direct mechanistic attribution; all mechanistic interpretations remain hypothesis-generating. The differential visit interval (every 3–4 days for cTOT vs. daily for HBOT and SOC) imposes a surveillance bias whose direction disfavors cTOT—less-frequent visits cause healing events to be detected at the next scheduled assessment rather than at their actual occurrence, biasing cTOT healing time upward; the observed treatment effect is therefore conservative under this bias. cTOT device adherence was assessed indirectly through outpatient attendance (96.3%) rather than objective usage logging.

Generalizability is limited to implant-based reconstruction with ADM; applicability to autologous reconstructions is unknown. Relatedly, our institutional NAC necrosis incidence of 30.5% (318 of 1043 NSMs over 2020–2025) exceeds the 5–30% range commonly reported in published NSM series [4,5]; this reflects the oncologically driven mastectomy-flap-thinness practice at our institution, in which the breast-oncology team deliberately leaves a thin mastectomy flap to minimize residual breast tissue and reduce oncological recurrence risk, at the trade-off cost of higher NAC ischemia rates. This institutional practice may limit generalizability to centers using thicker mastectomy flap protocols. Grade 3 necrosis was excluded as these patients typically require immediate surgical excision. Small subgroup sizes for current smokers (*n* = 11) and diabetic patients (*n* = 14) preclude reliable estimation. The 12-week follow-up captured healing but not long-term aesthetic results—which dedicated post-NSM fat-transfer reconstruction protocols have characterized over multi-year horizons [30]—or nipple sensitivity. We emphasize that these findings should be interpreted as hypothesis-generating Level III evidence that establishes clinical equipoise and justifies—but does not replace—a definitive multicenter randomized controlled trial. As noted in the Methods, intraoperative flap thickness was not routinely measured; post hoc surgeon inspection of representative ischemic cases suggested a flap thickness of approximately 5 mm or less, but this institutional practice was not quantitatively characterized within the present dataset and remains a methodological limitation.

Clinical implications. Acknowledging the limitations of retrospective evidence, these findings offer provisional guidance. For NAC necrosis involving larger wounds (≥ 2.0 cm^2^) or Grade 2 necrosis—where the risk of adjuvant delay and secondary surgery is highest—cTOT may be preferentially considered as first-line therapy given its strong treatment association, home-based administration, and absence of HBOT’s logistical constraints. For smaller Grade 1 wounds, standard care may be considered initially in resource-constrained settings; cTOT still demonstrated a significant benefit in this subgroup (HR 3.00). Where cTOT is unavailable, HBOT remains a viable alternative (HR 2.26). These recommendations should be considered hypothesis-generating, pending randomized validation.

Future directions. A multicenter randomized controlled trial comparing cTOT, HBOT, and standard care—powered for adjuvant delay-free survival and with a sham ODS comparator beneath standardized Mepilex Border Flex dressing—represents the logical next step to establish Level I evidence. Mechanistic studies should incorporate serial TcPO_2_, ICG angiography, and objective device-usage logging. The significant wound–area interaction suggests a potential role for prophylactic cTOT in at-risk mastectomy flaps identified by intraoperative perfusion assessment. A supplementary flexible-parametric survival model with time-varying coefficients revealed peak cTOT effect during weeks 2–3, attenuating by weeks 6–8 (Supplementary Figure S5). Extended indications including mastectomy skin-flap necrosis and free-flap compromise warrant investigation. Formal health economic modeling using Markov decision analysis would inform reimbursement policy. Emerging adjunctive topical wound therapies, such as nitric oxide-releasing gels under investigation for diabetic and other chronic wounds [31], also warrant comparative evaluation in this indication.

## 5. Conclusions

In this propensity-matched study of 213 patients—the largest cohort and first three-way comparison for this indication—cTOT was associated with accelerated NAC necrosis healing following NSM. The co-primary metrics—RMST difference of 14.0 days (95% CI, 10.5–17.5) and Cox HR 4.61 (95% CI, 2.99–7.11)—demonstrated a consistent treatment association across seven sensitivity analyses, with standardized dressing protocols (Mepilex Border Flex in all arms) ensuring that the principal between-group difference was oxygen delivery. E-values and interrupted time series modeling provided evidence against residual confounding and era effects as plausible alternative explanations. Secondary outcomes—including adjuvant therapy delay and secondary surgery rates—showed consistent trends favoring cTOT; analysis of adjuvant initiation as a continuous outcome yielded a significant 8.2-day reduction (*p* = 0.004), though binary endpoints did not reach statistical significance and require confirmation in adequately powered multicenter randomized trials. As a portable, home-based modality, cTOT offers a promising means to integrate wound management with oncological timelines—ensuring that the reconstructive benefits of NSM are not undermined by preventable delays in cancer therapy.

## Figures and Tables

**Table 1:** Baseline demographics and clinical characteristics (full cohort, *n* = 213). Among the 213 included patients, mean initial NAC involvement (necrosis area/total areolar surface area × 100) was 21.6% (range 2.8–80.4%). Lesion topography was predominantly nipple-centered (*n* = 144, 67.6%; mean NAC involvement 14.1%); a smaller proportion involved nipple plus partial areola (*n* = 59, 27.7%; mean NAC involvement 33.4%); NAC-extensive involvement (≥ 50% of the NAC) was rare (*n* = 10, 4.7%; mean NAC involvement 58.6%). This distribution confirms that the cohort comprises predominantly partial-thickness, nipple-centered necrosis rather than full NAC involvement, consistent with the spectrum reported in prior NSM series.

Variable	SOC	HBOT	cTOT	*p* Value	Max SMD	
	**(** ***n*** ** = 97)**	**(** ***n*** ** = 59)**	**(** ***n*** ** = 57)**		***Pre***	***Post***
Age, years, mean ± SD	48.3 ± 9.1	47.8 ± 8.7	49.2 ± 8.4	0.681	0.112	0.058
BMI, kg/m^2^, mean ± SD	24.3 ± 3.2	23.9 ± 2.9	24.7 ± 3.0	0.384	0.138	0.072
Smoking status, *n* (%)				0.714	0.184	0.091
*Never*	72 (74.2)	45 (76.3)	44 (77.2)			
*Former*	14 (14.4)	8 (14.3)	6 (11.1)			
*Current*	11 (11.3)	6 (10.2)	7 (12.3)			
Diabetes mellitus, *n* (%)	18 (18.6)	6 (10.2)	8 (14.0)	0.392	0.328	0.065
Hypertension, *n* (%)	29 (29.9)	10 (16.9)	11 (19.3)	0.175	0.478	0.082
Prior radiation, *n* (%)	8 (8.2)	5 (8.5)	3 (5.3)	0.766	0.108	0.044
Neoadjuvant chemotherapy, *n* (%)	35 (36.1)	22 (37.3)	18 (31.6)	0.793	0.087	0.053
Tumor location, *n* (%)				0.648	0.142	0.068
*Upper outer quadrant*	38 (39.2)	25 (42.4)	22 (38.6)			
*Upper inner quadrant*	22 (22.7)	16 (27.1)	16 (28.1)			
*Lower quadrants*	21 (21.6)	10 (17.9)	12 (22.2)			
*Central/multifocal*	16 (16.7)	8 (14.3)	7 (13.0)			
Tumor–nipple distance, cm	2.8 ± 1.1	2.4 ± 0.9	2.3 ± 1.0	0.023 *	0.404	0.076
Cancer stage, *n* (%)				0.542	0.156	0.088
*0 (DCIS)*	15 (15.5)	10 (17.9)	8 (14.8)			
*I*	42 (43.3)	29 (49.2)	30 (52.6)			
*II*	31 (32.0)	16 (27.1)	15 (26.3)			
*III*	9 (9.3)	4 (7.1)	4 (7.4)			
Mastectomy specimen weight, g	362.4 ± 118.7	378.5 ± 124.3	385.2 ± 115.6	0.449	0.168	0.074
Implant size, cc, mean ± SD	325.8 ± 54.2	318.6 ± 49.8	330.4 ± 52.1	0.498	0.147	0.062
ADM type, *n* (%)				0.856	0.092	0.048
*AlloDerm*	52 (53.6)	33 (55.9)	30 (52.6)			
*MegaDerm*	45 (46.4)	26 (44.1)	27 (47.4)			
Necrosis grade, *n* (%)				0.283	0.214	0.078
*Grade 1 (<50% NAC*)	55 (56.7)	30 (50.8)	27 (47.4)			
*Grade 2 (≥50% NAC*)	42 (43.3)	29 (49.2)	30 (52.6)			
Initial necrosis area, cm^2^	1.82 ± 0.94	1.96 ± 0.88	2.04 ± 0.91	0.341	0.198	0.064
Treatment initiation, POD	5.4 ± 1.6	5.2 ± 1.3	5.5 ± 1.5	0.520	—	—

Data are presented as mean ± SD or *n* (%). SMD, standardized mean difference; Pre, before propensity score matching; Post, after 1:1:1 matching (57 triplets, 171 patients). * Statistically significant (*p* < 0.05). All post-matching SMDs < 0.1, confirming excellent covariate balance. POD, postoperative day.

**Table 2:** Primary and secondary outcomes (matched cohort, *n* = 171).

Outcome	cTOT	HBOT	SOC	*p*	cTOT vs. SOC
	(*n* = 57)	(*n* = 57)	(*n* = 57)		*Difference*
*Primary outcome*					
Healing time, days, mean ± SD	30.5 ± 9.9	36.4 ± 9.0	45.1 ± 10.6	<0.001	−14.0 days
*Median (IQR)*	31 (23–38)	35 (30–42)	45 (38–50)		
*% reduction vs. SOC*	32.4%	19.3%	Ref.		
RMST at 56 days, days (95% CI)	48.0 (45.9–50.1)	43.5 (41.1–45.9)	34.0 (31.2–36.8)	<0.001	14.0 (10.5–17.5)
8-week healing rate, *n* (%)	56 (98.2)	55 (96.5)	50 (87.7)	<0.001	
*Wound trajectory*					
PAR at Week 2, %, mean ± SD	45.4 ± 7.5	41.0 ± 8.9	34.7 ± 8.2	<0.001	
PAR at Week 4, %, median	100.0	80.2	62.8	<0.001	
Residual area Week 4, cm^2^, median	0.00	0.32	1.14	<0.001	
*Secondary outcomes*					
Adjuvant delay > 6 weeks, *n* (%)	3 (5.3)	3 (5.3)	8 (14.0)	0.262	ARR 8.7 pp
Time to adjuvant, days, median (IQR)	35 (28–42)	38 (30–46)	44 (33–56)	0.008	−8.2 days
Secondary surgery, *n* (%)	3 (5.3)	7 (12.3)	11 (19.3)	0.132	NNT = 7
Implant loss, *n* (%)	0 (0.0)	1 (1.8)	1 (1.8)	0.384	
Wound infection, *n* (%)	2 (3.5)	3 (5.3)	7 (12.3)	0.258	
Pain VAS Week 2, mean ± SD	2.8 ± 1.2	3.8 ± 1.4	4.7 ± 1.8	<0.001	−1.9
Patient satisfaction, mean ± SD	8.0 ± 1.1	7.2 ± 1.3	5.7 ± 1.8	<0.001	+2.3

RMST, restricted mean survival time; PAR, percentage area reduction; VAS, visual analogue scale; IQR, interquartile range; ARR, absolute risk reduction; pp, percentage points; NNT, number needed to treat. *p* values from ANOVA (continuous), log-rank (time-to-event), or Fisher exact test (categorical). Pairwise comparisons: cTOT vs. SOC *p* < 0.001; cTOT vs. HBOT *p* = 0.007; HBOT vs. SOC *p* < 0.001 (Bonferroni-adjusted α = 0.017).

**Table 3:** Multivariable Cox proportional hazards regression for time to complete epithelialization (matched cohort, *n* = 171).

Variable	HR	95% CI	*p* Value	Schoenfeld P
Treatment group (ref: SOC)				
*cTOT*	4.61	2.99–7.11	<0.001	0.022
*HBOT*	2.26	1.51–3.38	<0.001	0.341
Necrosis grade (ref: Grade 1)				
*Grade 2*	2.08	1.29–3.35	0.001	0.487
Initial wound area, per cm^2^	0.76	0.59–0.97	0.005	0.612
Age, per year	0.99	0.97–1.01	0.382	0.754
BMI, per kg/m^2^	0.97	0.93–1.02	0.218	0.893
Current smoker (ref: never)	0.74	0.42–1.31	0.298	0.523
Diabetes mellitus	0.82	0.51–1.33	0.421	0.668
Neoadjuvant chemotherapy	0.91	0.64–1.30	0.608	0.445
Mean visit interval, days	1.03	0.88–1.21	0.714	0.382

HR > 1.0 indicates faster healing. Model C-statistic = 0.72. Global proportional hazards test *p* = 0.118 (no violation). Robust (sandwich) variance estimators used for matched clusters. Backward elimination with *p* < 0.10 retention threshold. The marginally significant Schoenfeld P for cTOT (0.022) indicates a time-dependent effect consistent with front-loaded benefit; the co-primary RMST analysis addresses this non-proportionality.

**Table 4:** Subgroup analysis: cTOT vs. standard care (matched cohort).

Subgroup	*n*	HR	95% CI	*p* Value	*p* Interaction
Overall	114	4.61	2.99–7.11	<0.001	—
Necrosis grade					*p* = 0.148
*Grade 1* (<50% *NAC*)	70	2.93	1.73–4.97	<0.001	
*Grade 2* (*≥50% NAC*)	44	8.81	4.04–19.20	<0.001	
Initial wound area					*p* = 0.018 *
*<2.0* cm^2^	73	3.00	1.81–4.99	<0.001	
*≥2.0* cm^2^	41	6.85	3.07–15.30	<0.001	
BMI					0.412
*<25* kg/m^2^	59	7.01	3.48–14.09	<0.001	
*≥25* kg/m^2^	55	2.67	1.51–4.74	<0.001	
Smoking status					0.547
*Never smoker*	85	3.38	2.09–5.47	<0.001	
*Ever smoker*	29	10.51	3.40–32.50	0.001	
Diabetes mellitus					0.624
*No*	107	3.56	2.33–5.44	<0.001	
*Yes*	7	—	too few events	—	
Cancer stage					0.358
*Stage 0–I*	57	4.66	2.44–8.92	<0.001	
*Stage II–III*	57	3.56	1.99–6.38	<0.001	

*n* represents cTOT + SOC patients in each subgroup from the matched cohort. HR > 1.0 favors cTOT (faster healing). * Significant interaction surviving Bonferroni correction (α = 0.008 for 6 comparisons). Small subgroup sizes for current smokers (*n* = 11) and diabetic patients (*n* = 14) preclude reliable estimation; these findings are exploratory.

## Data Availability

The datasets are not publicly available due to privacy and ethical restrictions but are available from the corresponding author on reasonable request.

## Supplementary Materials

The following supporting information can be downloaded at: https://www.mdpi.com/article/10.3390/cancers18121907/s1, Supplementary Methods S1: Surgical technique; Supplementary Methods S2: Clinical photography protocol; Supplementary Methods S3: Interrupted time series model specifications; Table S1: Sensitivity analysis results; Table S2: Competing risks analysis (matched cohort, *n* = 171); Table S3: IPTW-weighted analysis (full cohort, *n* = 213); Table S4: cTOT device-related adverse events (matched cohort, *n* = 57); Table S5: IPTW-weighted Cox regression (full cohort, *n* = 213); Table S6: Primary and secondary outcomes (full cohort, *n* = 213); Figure S1: cTOT device application protocol for NAC necrosis following nipple-sparing mastectomy. (A) The cTOT oxygen distribution system (ODS): A flexible silicone pad placed directly over the wound bed to deliver continuous pure humidified oxygen (>98%) to the wound surface. The ODS is connected to the generator via thin silicone tubing. (B) The portable cTOT electrochemical oxygen generator (~100 g, battery-powered), which produces pure humidified oxygen at ~15 mL/hour continuously (24 h/day, 7 days/week). The compact, lightweight design enables unrestricted home-based therapy and normal daily activities. (C) Clinical application of the cTOT system to a NAC necrosis wound following nipple-sparing mastectomy. The ODS is positioned directly over the wound bed, covered with a silicone foam dressing (Mepilex Border Flex, Mölnlycke Health Care) as the secondary dressing, and sealed with a transparent film dressing (Tegaderm™, 3M) to maintain the oxygen-enriched microenvironment beneath the foam. The generator is worn on the patient’s clothing or carried in a small pouch; Figure S2: Competing risks analysis (matched cohort, *n* = 171). (A) Cumulative incidence of complete epithelialization, treating secondary surgery as a competing event. cTOT (blue) demonstrates the highest cumulative incidence of healing across all time points. (B) Cumulative incidence of secondary surgery treating healing as a competing event. SOC (green) shows the highest cumulative incidence of secondary surgery. The Fine–Gray subdistribution hazard ratio for cTOT vs. SOC was 4.12 (95% CI, 2.68–6.34; *p* < 0.001), consistent with the primary cause-specific analysis; Figure S3: RMST 56-day area-under-curve visualization. Two-panel visualization of the restricted mean survival time (RMST) analysis (matched cohort, *n* = 171). (A) Cumulative incidence of complete epithelialization for cTOT (blue solid), HBOT (orange dashed), and SOC (green dash-dot); the area between the cTOT and SOC curves up to the 56-day window corresponds to the between-arm RMST difference. (B) Bar plot of RMST values (cTOT 48.0 d [95% CI 45.9–50.1], HBOT 43.5 d [41.1–45.9], SOC 34.0 d [31.2–36.8]). cTOT − SOC gain: 14.0 d (95% CI 10.5–17.5). cTOT − HBOT gain: 4.5 d; Figure S4: Secular trends in healing time by calendar year (2020–2025) and treatment group. Error bars represent 95% confidence intervals. No significant secular trend was observed (enrollment order *p* = 0.752 in interrupted time series analysis), indicating that the cTOT treatment association was not attributable to era effects or improvements in institutional wound care practices over time. SOC and HBOT were available throughout the study period (2020–2025); cTOT was introduced in September 2024; Figure S5: Schoenfeld residual plots for proportional hazards assessment (full cohort, *n* = 213). The red line represents the LOWESS smooth of scaled Schoenfeld residuals over time. A horizontal pattern indicates no violation of the proportional hazards assumption. The cTOT variable shows a downward trend consistent with a time-varying (front-loaded) treatment effect (Schoenfeld *p* = 0.022), indicating that the treatment benefit was most pronounced in the early weeks and attenuated as wounds approached complete healing. Other covariates (HBOT, Grade 2 necrosis, initial wound area, age, BMI) demonstrate approximately horizontal patterns consistent with proportional hazards. The global proportional hazards test was non-significant (*p* = 0.118). The co-primary RMST analysis, which does not depend on the proportional hazards assumption, was designated to address this non-proportionality.
