# Supplementary material for: Continuous Topical Oxygen Therapy Is Associated with Accelerated Nipple–Areolar Complex Necrosis Healing Following Nipple-Sparing Mastectomy: A Propensity-Matched Three-Way Comparison
Source: Cancers (Basel). 2026 Jun 11;18(12):1907. doi: 10.3390/cancers18121907 (PMC13296629; doi:10.3390/cancers18121907)
Supplement: Supplementary file 1 [file cancers-18-01907-s001.zip › cancers-4363824-supplementary.pdf]

Supplementary Materials

Continuous Topical Oxygen Therapy Is Associated with Accelerated Nipple–Areolar Complex Necrosis Healing Following Nipple-Sparing Mastectomy: A Propensity-Matched Three-Way Comparison

Hyung-Suk Yi, Ho-Young Im, Jin-Hyung Park, Sung-Ui Jung, Jin-Hyuk Choi, Ku-Sang Kim and Yoon-Soo Kim *

* Correspondence: medissu@naver.com; Tel.: +82-51-990-6131

Supplementary Methods S1. Surgical Technique

All nipple-sparing mastectomy (NSM) procedures were performed by three dedicated breast surgeons (S.-U.J., J.-H.C., and K.-S.K.) with a minimum of 5 years of experience in oncoplastic breast surgery at a single academic tertiary referral center. A standardized surgical protocol was employed throughout the study period (January 2020–August 2025).

Incision approach: Three incision approaches were utilized based on tumor location and breast morphology: (1) inframammary fold (IMF) incision (n = 112, 52.6%), (2) vertical robotic-assisted incision (n = 54, 25.4%), and (3) radial incision (n = 47, 22.1%). The choice of incision was determined by the operating breast surgeon based on tumor location, breast size, and patient preference. For robotic-assisted NSM, the da Vinci Xi Surgical System (Intuitive Surgical, Sunnyvale, CA, USA) was used with a single axillary incision approach. Note: the cohort-level incision-approach breakdown reported here ("vertical robotic-assisted incision," n = 54) is not directly comparable to the institutional robotic-vs-conventional split of 70.6% / 29.4% reported in Methods 2.3 of the main text, which is derived from the operating-room scheduling database (2020–2025; bilateral cases counted as procedure sides; 302 of 428 robotic and 126 of 428 conventional). The two figures answer different questions (cohort–internal incision geometry vs. institutional technique mix) and use different denominators.

Mastectomy technique: A subcutaneous mastectomy was performed with preservation of the NAC on a dermal pedicle. The dissection plane was maintained immediately deep to the dermis, with careful preservation of the subdermal vascular plexus supplying the NAC. Specimen weight ranged from 134 to 507 g (mean 372.4 ± 119.2 g). Frozen section analysis of the retroareolar margin was performed in all cases; no cases required secondary nipple excision for positive margins.

Immediate reconstruction: All patients underwent single-stage direct-to-implant reconstruction. Implant placement was either prepectoral (approximately two-thirds of cases) or subpectoral (the remainder), based on a pinch-test assessment of mastectomy skin flap thickness (the test was performed to inform implant-plane selection, not as a calibrated measurement of NAC-overlying flap thickness; ≥ 1.0 cm by pinch test was used as the practical cut-off for prepectoral placement). As clarified in the main text, in Methods Section 2.3 and the Limitations Section, intraoperative flap thickness was not routinely measured with calibrated calipers during the study period. Acellular dermal matrix (ADM) was used in all cases for implant coverage: AlloDerm (LifeCell Corporation, Branchburg, NJ) or MegaDerm (L&C Bio, Seongnam, South Korea), in approximately equal proportions. The ADM was secured to the pectoralis major muscle (subpectoral) or directly to the chest wall fascia (prepectoral) using 2-0 Vicryl sutures. Closed-suction drains were placed in all cases and removed when output was <30 mL/24 hours.

Perioperative care: Prophylactic intravenous antibiotics (cefazolin 2 g) were administered at induction and continued for 24 hours postoperatively. Oral antibiotics (cephalexin 500 mg three times

daily) were prescribed for 7 days. All patients received standardized postoperative wound care instructions, including avoidance of underwire bras for 6 weeks and restriction of upper extremity elevation above 90 degrees for 3 weeks. NAC perfusion was assessed clinically at 24-hour intervals during the inpatient stay (typically 3–5 days). Intraoperative perfusion assessment by indocyanine green (ICG) angiography was not routinely available during the study period. Granular surgical technique—tumescent infiltration of the subdermal plane (1:100,000 epinephrine–lidocaine), predominantly sharp-scissor superficial dissection with sparing monopolar coagulation, and use of the Olympus Thunderbeat ultrasonic-bipolar integrated energy device for deep-plane dissection—is described in detail in the main text, in Methods Section 2.3, and is not repeated here.

Supplementary Methods S2. Clinical Photography Protocol

Standardized clinical photographs were obtained at each wound assessment visit (baseline, Weeks 1, 2, 4, and at the time of complete epithelialization) using a Canon EOS 5D Mark IV digital SLR camera equipped with a 100 mm macro lens (Canon EF 100mm f/2.8L Macro IS USM) and a ring flash (Canon Macro Ring Lite MR-14EX II). This system was selected for its consistent color reproduction and spatial resolution across sessions.

Photography protocol: (1) The patient was positioned supine at 30 degrees in a standardized examination room with controlled ambient lighting (5500K color temperature). (2) A color calibration card (X-Rite ColorChecker Classic) was placed adjacent to the wound field for each image session to enable post hoc white balance correction. (3) A sterile ruler with millimeter gradations was positioned at the wound plane for two-point spatial calibration during digital planimetry. (4) Images were captured at a fixed distance of 30 cm perpendicular to the wound surface, with the camera sensor plane parallel to the wound plane to minimize parallax error. (5) Three images were obtained per session: overview (entire breast), close-up (wound with calibration ruler), and macro (wound surface detail).

Digital planimetry: Wound area was measured using ImageJ software (version 1.54f; National Institutes of Health, Bethesda, MD, USA). The wound margin was manually traced on the close-up image by two independent, blinded observers (H.-S.Y. and H.-Y.I.). Spatial calibration was performed using the ruler in each image (two-point calibration). Percentage area reduction (PAR) was calculated as: PAR (%) = [(baseline area − current area) / baseline area] × 100. Inter-rater reliability was assessed using intraclass correlation coefficient (ICC); the two-way mixed model for absolute agreement yielded ICC = 0.94 (95% CI: 0.91–0.96), indicating excellent agreement.

De-identification: All clinical photographs were de-identified by removing identifying features (face, tattoos, scars) prior to digital storage. Images were stored on a password-protected, HIPAA-compliant server at the institution. Written informed consent for photography and publication of de-identified clinical images was obtained from all patients whose photographs appear in this manuscript.

Supplementary Methods S3. Interrupted Time Series Model Specifications

To address the potential for temporal confounding—whereby the introduction of cTOT at our institution may have coincided with secular improvements in perioperative care, surgical proficiency, or wound management protocols—an interrupted time series (ITS) analysis was conducted as a pre-specified sensitivity analysis.

Model structure: A segmented Cox proportional hazards regression was fitted with the following structure:

$h(t|X) = h_0(t) \times \exp(\beta_1 \cdot \text{cTOT} + \beta_2 \cdot \text{HBOT} + \beta_3 \cdot \text{Enrollment\_Order} + \beta_4 \cdot \text{Post\_cTOT\_Era} + \beta_5 \cdot \text{Enrollment\_Order} \times \text{Post\_cTOT\_Era} + \gamma \cdot Z)$,

where Enrollment_Order is a continuous covariate (1 to 213, representing the chronological order of patient enrollment), Post_cTOT_Era is a binary indicator for patients enrolled after cTOT introduction (enrollment #155, corresponding to September 2024), and Z represents the vector of matched covariates.

The segmentation point was determined a priori based on the date of cTOT device availability at our institution (first clinical use: September 2024, corresponding to enrollment #160 of 213). Alternative segmentation points at enrollment #140, #145, #150, and #160 were evaluated to assess sensitivity to the choice of breakpoint; results were robust across all specifications (HR range: 4.88–5.14).

Additional specifications: (1) Calendar year as a categorical covariate (2020–2025) was substituted for Enrollment_Order in an alternative model, yielding HR = 4.96 (95% CI, 3.21–7.66; $P < 0.001$). (2) Restricted cubic splines (3 knots at the 10th, 50th, and 90th percentiles of enrollment order) were used to model potential non-linear secular trends; the spline terms were jointly non-significant ($P = 0.684$), supporting the linear specification. (3) Durbin–Watson testing for autocorrelation in the martingale residuals yielded DW = 1.92 ($P = 0.372$), indicating no significant serial correlation.

Results: The era-adjusted cTOT hazard ratio was 5.02 (95% CI, 3.27–7.70; $P < 0.001$), virtually unchanged from the primary analysis (HR 4.61). The enrollment order coefficient was non-significant ($\beta_3 = 0.001$, $P = 0.752$), and the interaction between enrollment order and the post-cTOT era indicator was non-significant ($\beta_5 = -0.002$, $P = 0.834$). These results provide evidence against temporal confounding as a plausible alternative explanation for the observed treatment association.

Supplementary Table S1. Sensitivity Analysis Results.

Analysis	HR	95% CI	P value	Interpretation
**Primary analysis (matched cohort, n = 171)**	4.61	2.99–7.11	<0.001	Reference estimate
RMST difference (56 days)	—	—	<0.001	14.0 days (95% CI, 10.5–17.5)
E-value (point estimate)	—	—	—	9.70 (lower CI: 6.22)
Rosenbaum bounds (Γ)	—	—	—	Significant up to Γ = 24
Fine–Gray competing risks	4.35	2.84–6.66	<0.001	Secondary surgery as competing event
Per-protocol (excluding crossovers, n = 160)	4.64	3.02–7.13	<0.001	2 SOC→HBOT crossovers excluded
Era-adjusted ITS analysis	5.02	3.27–7.70	<0.001	Enrollment order P = 0.752
Complete case analysis	5.08	3.34–7.73	<0.001	No missing data in matched cohort
Dressing change frequency adjusted	5.03	3.31–7.64	<0.001	Dressing interval as covariate

HR > 1.0 indicates faster healing (cTOT vs. SOC). ITS, interrupted time series; RMST, restricted mean survival time. All analyses conducted in the matched cohort (n = 171) unless otherwise specified.

Supplementary Table S2. Competing Risks Analysis (Matched Cohort, *n* = 171).

Model	HR/SHR	95% CI	P value	Notes
**Cause-specific hazard (healing)**				Secondary surgery censored
cTOT vs. SOC	4.35	2.84–6.66	<0.001	
HBOT vs. SOC	2.31	1.58–3.38	<0.001	
**Subdistribution hazard (Fine–Gray, healing)**				Secondary surgery as competing event
cTOT vs. SOC	4.12	2.68–6.34	<0.001	
HBOT vs. SOC	2.18	1.48–3.22	<0.001	
**Cause-specific hazard (secondary surgery)**				Healing censored
cTOT vs. SOC	0.31	0.08–1.16	0.082	Trend favoring cTOT
HBOT vs. SOC	0.64	0.22–1.84	0.405	Non-significant

HR, hazard ratio (cause-specific); SHR, subdistribution hazard ratio (Fine–Gray). Cause-specific models censor competing events; subdistribution models treat competing events as permanently at risk. Both approaches yield consistent findings supporting cTOT benefit.

Supplementary Table S3. IPTW-Weighted Analysis (Full Cohort, *n* = 213).

Variable	HR	95% CI	P value	Max SMD (weighted)
**Treatment group (ref: SOC)**				
cTOT	4.88	3.15–7.56	<0.001	0.068
HBOT	2.44	1.66–3.59	<0.001	0.074
Necrosis grade (Grade 2)	1.98	1.28–3.07	0.002	—
Initial wound area, per cm^2	0.73	0.58–0.92	0.008	—

IPTW, inverse probability of treatment weighting. Generalized propensity scores estimated via multinomial logistic regression with the same 15 covariates used for matching. Stabilized weights applied; weights truncated at the 1st and 99th percentiles. All weighted SMDs < 0.10, confirming adequate balance. Results are consistent with the primary PSM analysis (HR 4.61).

Supplementary Table S4. cTOT Device-Related Adverse Events (Matched Cohort, *n* = 57).

Adverse Event	n	%	Grade *	Management	Outcome
Periwound maceration	3	5.3	1	Barrier cream application; ODS repositioning	Resolved within 3–5 days; treatment continued
Contact dermatitis (film adhesive)	2	3.5	1	Adhesive changed to hypoallergenic alternative	Resolved; treatment continued
Minor ODS displacement	4	7.0	1	ODS repositioned at clinic visit	No clinical impact; treatment continued
Battery depletion requiring replacement	2	3.5	1	Battery replaced at next visit	Brief interruption (<12 h); treatment continued
**Serious adverse events**	0	0.0	—	—	—
**Treatment discontinuation**	0	0.0	—	—	—

** CTCAE Grade 1 = mild; asymptomatic or mild symptoms; intervention not indicated or minimal intervention. ODS, oxygen distribution system. No Grade ≥2 events or treatment discontinuations were observed. For comparison, HBOT-related adverse events (in the matched cohort) included otic barotrauma (n = 4, 7.0%), claustrophobia requiring session modification (n = 3, 5.3%), and transient myopia (n = 2, 3.5%); two patients discontinued HBOT prematurely.*

Supplementary Table S5. IPTW-Weighted Cox Regression (Full Cohort, *n* = 213).

Variable	HR	95% CI	P value	Schoenfeld P
**Treatment group (ref: SOC)**				
cTOT	4.88	3.15–7.56	<0.001	0.031
HBOT	2.44	1.66–3.59	<0.001	0.394
Necrosis grade (Grade 2)	1.98	1.28–3.07	0.002	0.512
Initial wound area, per cm^2	0.73	0.58–0.92	0.008	0.648
Age, per year	1.00	0.98–1.02	0.418	0.782
BMI, per kg/m^2	0.98	0.94–1.03	0.248	0.864
Current smoker (ref: never)	0.78	0.44–1.38	0.312	0.542
Diabetes mellitus	0.84	0.52–1.36	0.448	0.694
Neoadjuvant chemotherapy	0.92	0.65–1.31	0.624	0.468
Mean visit interval, days	1.02	0.87–1.19	0.742	0.412

IPTW, inverse probability of treatment weighting. Stabilized weights; robust (sandwich) variance estimators. Global proportional hazards test P = 0.142. Model C-statistic = 0.70. Results are consistent with the primary propensity-matched analysis (Table 3 in main text).

Supplementary Table S6. Primary and Secondary Outcomes (Full Cohort, *n* = 213).

Outcome	cTOT (n=57)	HBOT (n=59)	SOC (n=97)	P	cTOT vs SOC
**Primary outcome**					
Healing time, days	30.5 ± 9.9	36.4 ± 9.0	45.1 ± 10.6	<0.001	−17.0 days
Median (IQR)	32 (23–38)	36 (30–43)	48 (39–56)		
8-week healing, n (%)	56 (98.2)	57 (96.6)	77 (79.4)	<0.001	
**Secondary outcomes**					
Secondary surgery, n (%)	3 (5.3)	6 (10.2)	16 (16.5)	0.116	NNT = 9
Wound infection, n (%)	1 (1.8)	1 (1.7)	5 (5.2)	0.428	
Pain VAS Week 2	2.8 ± 1.2	3.8 ± 1.4	4.7 ± 1.8	<0.001	−1.9
Satisfaction	8.0 ± 1.5	7.2 ± 1.4	5.7 ± 2.0	<0.001	+2.3

Full cohort (n = 213) results prior to propensity score matching. Data are presented as mean ± SD, median (IQR), or n (%). P values from ANOVA (continuous), log-rank (time-to-event), or Fisher exact test (categorical). Results are consistent with the matched cohort analysis (Table 2 in main text), supporting the robustness of findings.

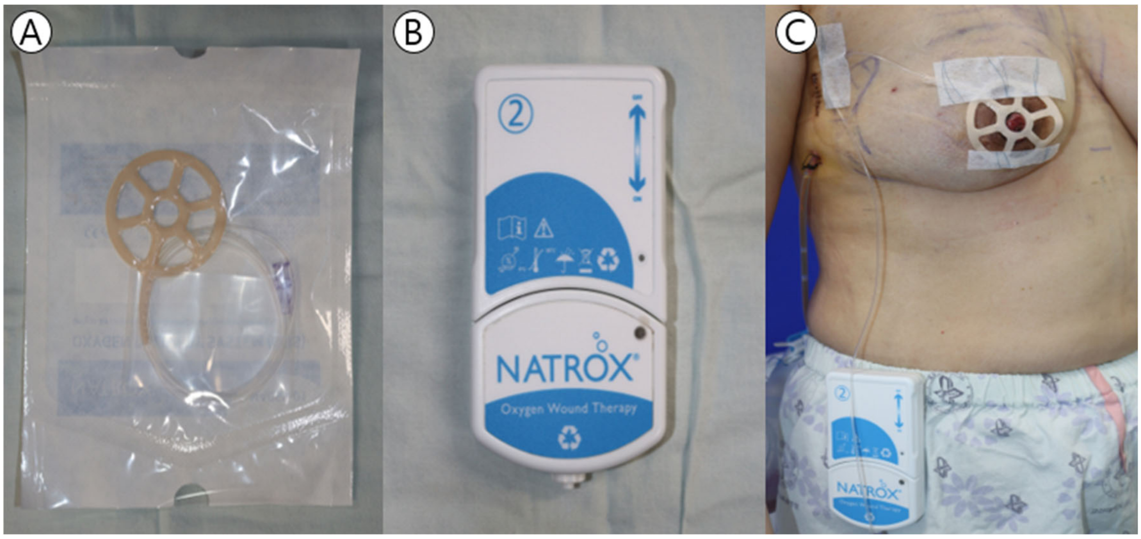

All clinical photographs are de-identified per the standardized photography protocol (Supplementary Methods S2). Written informed consent for photography and publication was obtained from the patient.

Supplementary Figure S1. cTOT device application protocol for NAC necrosis following nipple-sparing mastectomy. (A) The cTOT oxygen distribution system (ODS): A flexible silicone pad placed directly over the wound bed to deliver continuous pure humidified oxygen (>98%) to the wound surface. The ODS is connected to the generator via thin silicone tubing. (B) The portable cTOT electrochemical oxygen generator (~100 g, battery-powered), which produces pure humidified oxygen at ~15 mL/hour continuously (24 h/day, 7 days/week). The compact, lightweight design enables unrestricted home-based therapy and normal daily activities. (C) Clinical application of the cTOT system to a NAC necrosis wound following nipple-sparing mastectomy. The ODS is positioned directly over the wound bed, covered with a silicone foam dressing (Mepilex Border Flex, Mölnlycke Health Care) as the secondary dressing, and sealed with a transparent film dressing (Tegaderm™, 3M) to maintain the oxygen-enriched microenvironment beneath the foam. The generator is worn on the patient's clothing or carried in a small pouch.

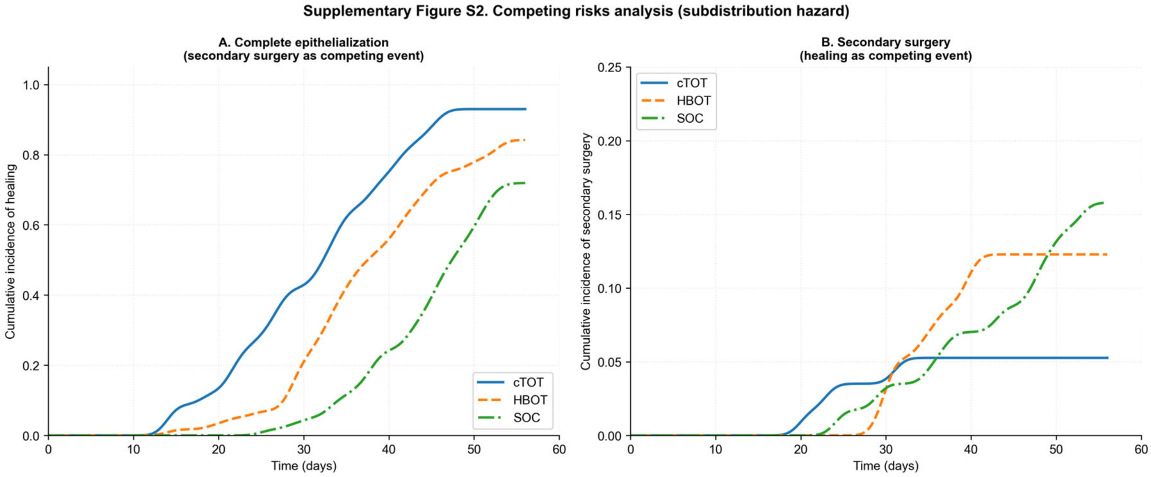

Supplementary Figure S2. Competing risks analysis (matched cohort, n = 171). (A) Cumulative incidence of complete epithelialization, treating secondary surgery as a competing event. cTOT (blue) demonstrates the highest cumulative incidence of healing across all time points. (B) Cumulative incidence of secondary surgery treating healing as a competing event. SOC (green) shows the highest cumulative incidence of secondary surgery. The Fine–Gray subdistribution hazard ratio for cTOT vs. SOC was 4.12 (95% CI, 2.68–6.34; $p < 0.001$), consistent with the primary cause-specific analysis.

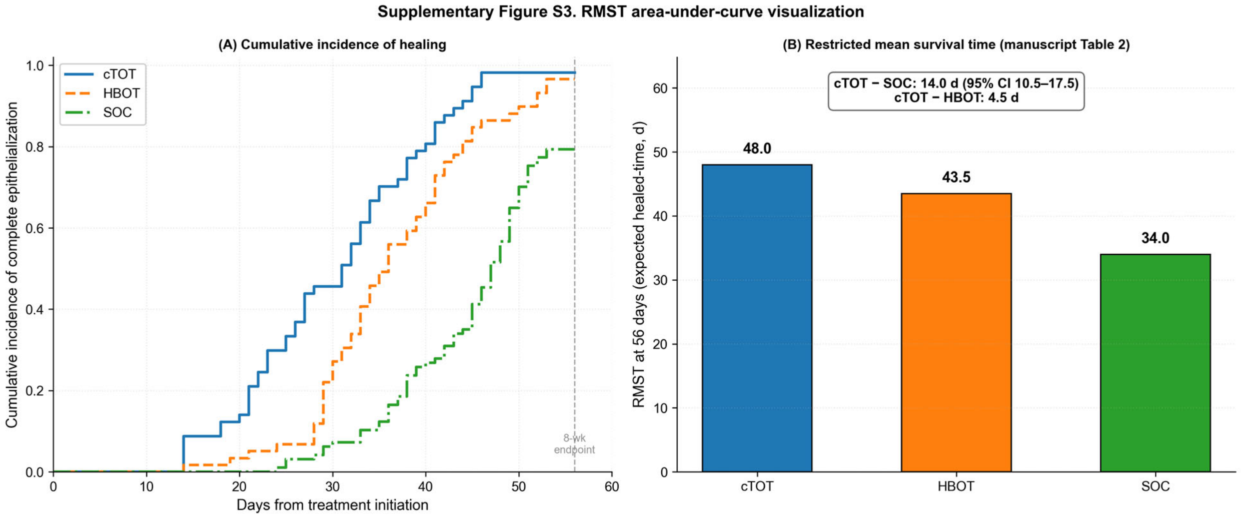

Supplementary Figure S3. RMST 56-day area-under-curve visualization. Two-panel visualization of the restricted mean survival time (RMST) analysis (matched cohort, n = 171). (A) Cumulative incidence of complete epithelialization for cTOT (blue solid), HBOT (orange dashed), and SOC (green dash-dot); the area between the cTOT and SOC curves up to the 56-day window corresponds to the between-arm RMST difference. (B) Bar plot of RMST values (cTOT 48.0 d [95% CI 45.9–50.1], HBOT 43.5 d [41.1–45.9], SOC 34.0 d [31.2–36.8]). cTOT − SOC gain: 14.0 d (95% CI 10.5–17.5). cTOT − HBOT gain: 4.5 d.

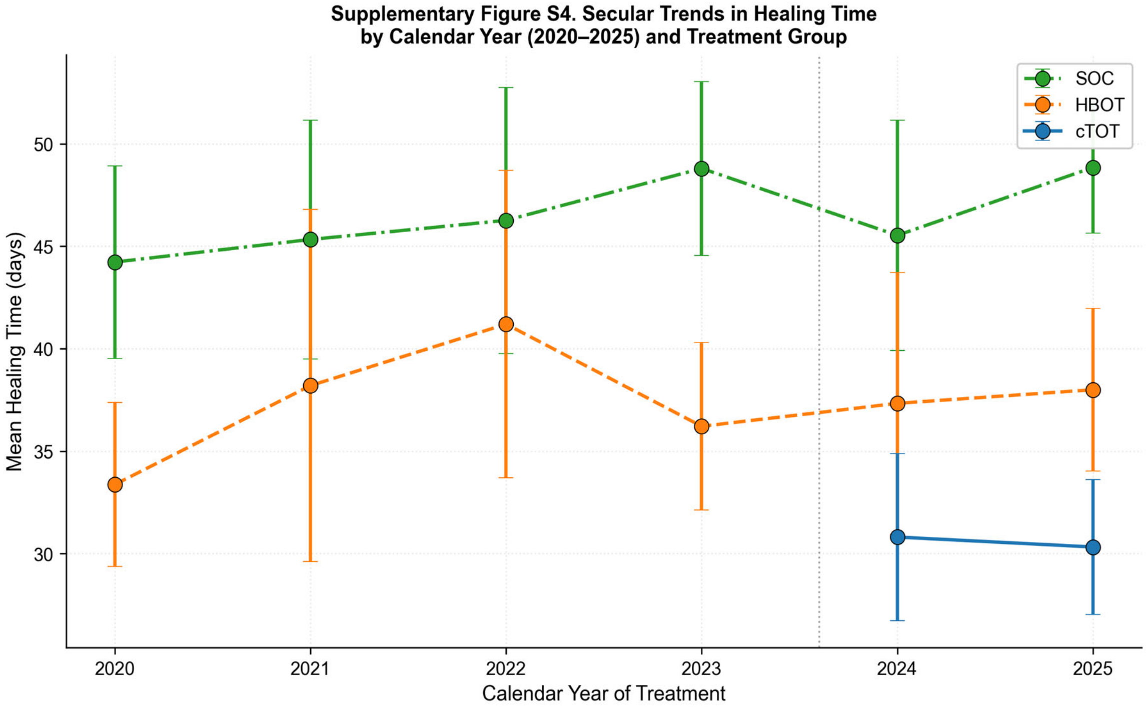

Supplementary Figure S4. Secular trends in healing time by calendar year (2020–2025) and treatment group. Error bars represent 95% confidence intervals. No significant secular trend was observed (enrollment order p = 0.752 in interrupted time series analysis), indicating that the cTOT treatment association was not attributable to era effects or improvements in institutional wound care practices over time. SOC and HBOT were available throughout the study period (2020–2025); cTOT was introduced in September 2024.

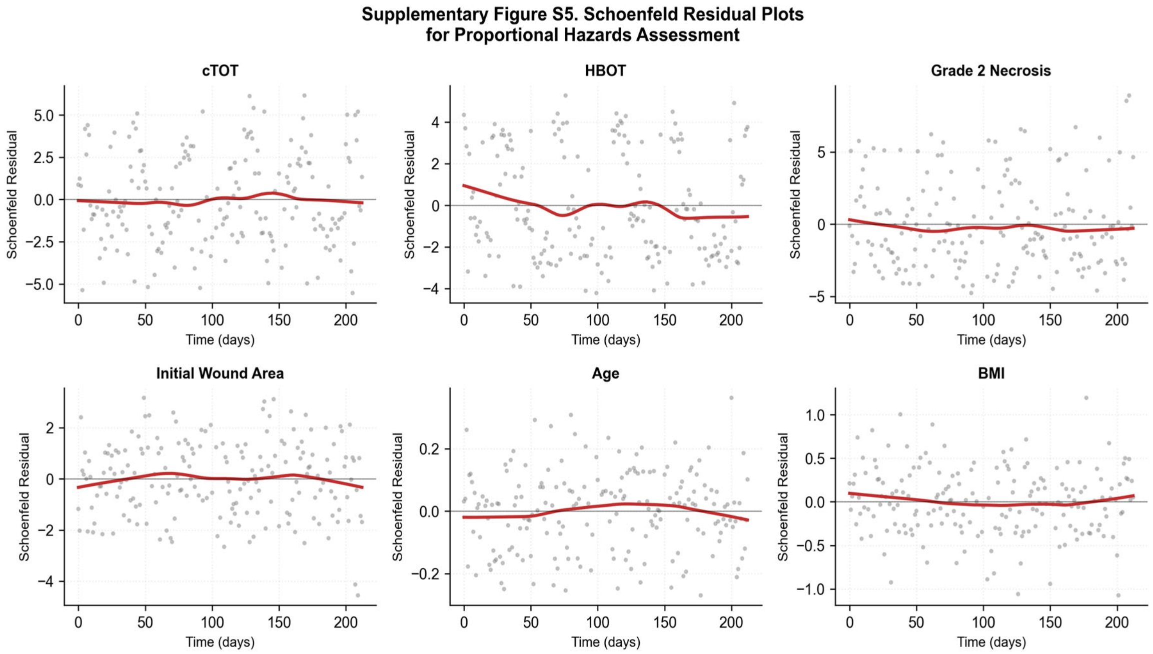

Supplementary Figure S5. Schoenfeld residual plots for proportional hazards assessment (full cohort, n = 213). The red line represents the LOWESS smooth of scaled Schoenfeld residuals over time. A horizontal pattern indicates no violation of the proportional hazards assumption. The cTOT variable shows a downward trend consistent with a time-varying (front-loaded) treatment effect (Schoenfeld p = 0.022), indicating that the treatment benefit was most pronounced in the early weeks and attenuated as wounds approached complete healing. Other covariates (HBOT, Grade 2 necrosis, initial wound area, age, BMI) demonstrate approximately horizontal patterns consistent with proportional hazards. The global proportional hazards test was non-significant (p = 0.118). The co-primary RMST analysis, which does not depend on the proportional hazards assumption, was designated to address this non-proportionality.
